# Vascular Calcification and Inherited Real Risk of Lithiasis

**DOI:** 10.3389/fendo.2012.00119

**Published:** 2012-10-05

**Authors:** Sergio Stagnaro, Simone Caramel

**Affiliations:** ^1^International Society of Quantum Biophysical SemeioticsLancenigo, Italy

## Introduction

We consider your paper really fascinating and written in a refined manner. Compliment. Unfortunately, under the same environmental conditions, not all subjects develop vascular calcification! The *condition sine qua non* also of vascular calcification is “Quantum Biophysical Semeiotics” (QBS) lithiasic Constitution-Dependent “Inherited Real Risk.”

## The “Inherited Real Risk” of Lithiasis

“Quantum Biophysics Semeiotics” (QBS) (Stagnaro and Stagnaro-Neri, 2004a) is a new discipline in the medical field and an extension of the classical semeiotics with the support of quantum and complexity theories. It is a scientific trans-disciplinary approach that is based on the “Congenital Acidosic Enzyme-Metabolic Histangiopathy” (CAEMH), a unique mitochondrial cytopathy that is present at birth and subject to medical therapy.

The presence of intense CAEMH in a well-defined area (i.e., myocardium) is due to gene mutations in both n-DNA and mit-DNA. This is the basis for one or more QBS constitutions which could bring about their respective Inherited Real Risks (IRR). IRR are characterized by microcirculatory remodeling, especially intense under environmental risk factors, showing typical type I, subtype (a) oncological and/or (b) aspecific “Endoarteriolar Blocking Devices” (EBD), as described and illustrated previously (Stagnaro, 2009).

The QBS method allows the clinical and pre-clinical diagnosis of the most severe diseases, e.g., solid and liquid forms of cancer, Type II Diabetes Mellitus and coronary heart diseases, as well as the IRR of lithiasis; i.e., through the auscultatory percussion of the stomach (Stagnaro-Neri and Stagnaro, 1988).

Doctors can now evaluate, with a stethoscope and the auscultation of any viscera (i.e., stomach, ureter), mitochondria functions as well as the behavior of any biological system. The presence of the IRR of many diseases linked with “QBS Constitutions” (i.e., lithiasic “QBS Constitution,” Stagnaro and Stagnaro-Neri, 1990; Stagnaro-Neri and Stagnaro, 1993b), is clinically diagnosable from birth so that an intelligent prevention strategy can be implemented only on those at real risk (with IRR of any disease).

In health, without any “risk” for stones (i.e., in absence of the “variant” Reaven’s syndrome (Stagnaro-Neri and Stagnaro, 1993a), the auscultation of the stomach under an intense digital pressure applied upon the projected skin area of the gall-bladder (i.e., right superior abdominal quadrant) does not provoke a simultaneous gastric dilation and the contraction of the antral-pyloric region. This sign is termed “Gastric Aspecific Reflex” (GAR).

In subjects with lithiasic Constitution, the above mentioned maneuver brings about a simultaneous GAR; i.e., the stomach dilates and then there is a small tonic gastric contraction (just a little if there is just the IRR of disease and very significant in case of overt lythiasis).

When lithiasic Constitution is recognized (Stagnaro and Stagnaro-Neri, 2004b, 2005), we should refine the diagnosis in order to determine the severity of the metabolic (lithiasis in progress) or pre-metabolic (grade of evolution of IRR of lithiasis) syndrome.

In health, a mean intensity digital pressure applied upon the above mentioned trigger points, brings about the GAR after a latency time of exactly 8 s, while the intensity is less than 2 cm. The reflex lasts for less than 4 s and then it disappears (disappearance time is physiologically between 3 and 4 s) before the next one appears.

On the contrary, in case of even clinically silent gall-bladder stone(s), the latency time of GAR is shorter than before (i.e., 3–4 s), while the intensity is 2 cm or more (in relation to the disorder entity). Soon thereafter, characteristically its intensity reduces rapidly to 1/3 of its greatest intensity: it is termed “lithiasic reflex” (Stagnaro, 2007). This sign is observed also during stimulation of specific trigger points, in case of other stones and tissue calcium deposition, localized in any biological system (i.e., kidneys and arterial wall).

This gastric diagnosis is consistent and dually reflects the informative nature and quality of parameters collected by QBS microcirculatory investigations that are in accord with clinical microangiology. The patho-physiology of QBS reflexes is based upon local microvascular conditions, i.e., of the gall-bladder wall.

In case of genetic alteration of both DNAs, there is a microcirculatory remodeling due to vasomotility and vasomotion impairment (e.g., functional imperfection) and structural obstructions, i.e., Arteriovenous Anastomosis (AVA) and EBD. In presence of lithiasic IRR, a slight digital pressure upon the above mentioned trigger points brings about a upper ureteral reflex (due to tissue acidosis), related to hepatic microcirculatory activation, even at rest (both vasomotion and vasomotility showing 7 s of diastole and 5 s of systole, instead of the basal 6 + 6).

## CAD, Vascular Calcification and Lithiasis Reflex

With the aid of QBS “Myocardial Ischemic Preconditioning” and the cardio-lithiasic GAR, one of the authors examined 150 individuals positive for Coronary Artery Disease (CAD) (Stagnaro-Neri and Stagnaro, 1997; Stagnaro, 2003). In these subjects, a digital pressure of mean intensity applied precisely upon the projected skin area of the calcified coronary, brings about cardio-lithiasic GAR, which, after reaching its highest value, immediately reduces to 1/3 of intensity: typical lithiasis reflex.

Interestingly, lithiasis exclusively involves individuals with “variant” metabolic syndrome. Therefore, coronary calcification are exclusively present in a subtype of patients involved by CAD: without Metabolic Syndrome “variant form,” calcifications are not possible!

In all 150 patients with CAD, even asymptomatic, latency time (myocardial oxygenation) became clearly shorter than that of the pathological basal value, in relation to severity of CAD. In addition, as noted by one of the authors, lithiasic GAR was observed in 48 cases with varying intensity. Diagnosis was subsequently corroborated with sophisticated semeiotics. In apparently healthy individuals, but diagnosed with IRR of CAD, reflex duration is longer than 4 s.

## Conclusion

For the first time, QBS allows doctors to clinically diagnose patients with IRR of CAD, overt CAD, symptomless of CAD and coronary artery calcification, years or decades before the disorder’s onset. This allows for proper primary and pre-primary prevention, i.e., through the “blue therapy” (Stagnaro and Caramel, 2011). Coronary artery calcification diagnosis is independent of its size and location, which can be today ascertained by means of EBCT. Interestingly, lithiasis in whichever biological system or tissue exclusively involves individuals with “variant” metabolic syndrome, as described previous (Stagnaro-Neri and Stagnaro, 1993a).
